# Supplementary material for: Dynamic physical examination indicators of cardiovascular health: A single-center study in Shanghai, China
Source: PLoS One. 2022 May 12;17(5):e0268358. doi: 10.1371/journal.pone.0268358 (PMC9098044; doi:10.1371/journal.pone.0268358)
Supplement: Attachment — Submitted filename: plos-Wenya Yu.docx [file pone.0268358.s004.docx]

The study by Kuang et al. attempted to to analyze dynamic physical examination indicators for cardiovascular health to provide evidence for the precise prevention and control of cardiovascular diseases in the primary prevention domain among healthy individuals with different demographic characteristics in Shanghai. and if so, to provide more accurate evidence for health providers to prevent and control CVDs in healthy individuals, especially in high-risk populations.

The authors reported that The CVH status of healthy individuals in Shanghai demonstrated a decreasing trend from 2018 to 2020. Strengthening the primary prevention of CVD-related physical examination indicators among high-risk individuals, especially for the dynamic change in trends of SBP, DBP, BMI, and levels of TG, LDL-C, and Glu in men, as well as the change in trend of TC levels in women, is necessary. Moreover, healthcare providers should closely monitor the CVH indicators in people aged 50–69 years, as these indicators are more sensitive to controlling the prevalence of CVDs in this population.

1. Incremental findings: Previous studies have reported that increasing SBP, DBP, BMI, and TG, LDL-C, and Glu levels and decreasing HDL-C levels increases the risk of CVDs. Combined with the results of this study, the SBP, DBP, BMI, and TG, LDL-C, and Glu levels of men were higher than those of women for each year, while their HDL-C levels were lower than those of women. Therefore, these findings will provide explicit evidence for early identification and timely intervention in high-risk male populations based on the changing trend of the indicators mentioned above.
2. Dietary habits, exercise habits, and sleep characteristics were not enrolled.
3. Analysis done between male and female group.
4. The manuscript readability was improved.
